# Integrating Equity Into Bicycle Infrastructure, Planning, and Programming: A Mixed Methods Exploration of Implementation Among Participants in the Bicycle Friendly Community Program

**DOI:** 10.5888/pcd20.230119

**Published:** 2023-10-05

**Authors:** Stephenie C. Lemon, Amelia Neptune, Melissa Goulding, Jyothi Ananth Pendharkar, Roddrick Dugger, Jamie F. Chriqui

**Affiliations:** 1 Division of Preventive and Behavioral Medicine, Department of Population and Quantitative Health Sciences, University of Massachusetts Chan Medical School, Worcester, Massachusetts; 2 League of American Bicyclists, Washington, District of Columbia; 3 Morningside Graduate School of Biomedical Sciences, University of Massachusetts Chan Medical School, Worcester, Massachusetts; 4 Arnold School of Public Health, University of South Carolina, Columbia, South Carolina; 5 School of Public Health, University of Illinois, Chicago, Illinois

## Abstract

**Introduction:**

Integrating equity considerations into bicycle infrastructure, planning, and programming is essential to increase bicycling and reduce physical inactivity–related health disparities. However, little is known about communities’ experiences with activities that promote equity considerations in bicycle infrastructure, planning, and programming or about barriers and facilitators to such considerations. The objective of this project was to gain in-depth understanding of the experiences, barriers, and facilitators that communities encounter with integrating equity considerations into bicycle infrastructure, planning, and programming.

**Methods:**

We administered a web-based survey in 2022 to assess communities’ experiences with 31 equity-focused activities in 3 areas: 1) community engagement, education, events, and programming (community engagement); 2) data collection, evaluation, and goal setting (data); and 3) infrastructure, facilities, and physical amenities (infrastructure). Respondents were people who represented communities in the US that participated in the League of American Bicyclists’ Bicycle Friendly Community (BFC) Program. We then conducted 6 focus groups with a subset of survey respondents to explore barriers and facilitators to implementing equity-focused activities.

**Results:**

Survey respondents (N = 194) had experience with a mean (SD) of 5.9 (5.7) equity-focused activities. Focus group participants (N = 30) identified themes related to community engagement (outreach to and engagement of underrepresented communities, cultural perceptions of bicycling, and funding and support for community rides and programs); data (locally relevant data); and infrastructure (political will, community design, and infrastructure). They described barriers and facilitators for each.

**Conclusion:**

Communities are challenged with integrating equity into bicycle infrastructure, planning, and programming. Multicomponent strategies with support from entities such as the BFC program will be required to make progress.

**Summary**

**What is already known about this topic?**

Bicycle infrastructure, planning, and programming can promote physical activity in communities.

**What is added by this report?**

We conducted a mixed methods study with representatives of communities who participated in the League of American Bicyclists’ Bicycle Friendly Community program to gain an in-depth understanding of the experiences, barriers, and facilitators that communities encounter when integrating equity considerations in bicycle infrastructure, planning, and programming.

**What are the implications for public health practice?**

Integrating equity considerations in community bicycle infrastructure, planning, and programming will require coordinated efforts that include strategies to build data capacity, community engagement, buy-in, and political will.

## Introduction

Bicycling, whether for transportation, leisure, or sport, is a potential way to achieve recommended levels of physical activity and reduce physical inactivity–associated disease of risk and death (1). Bicycling as a means of transportation brings additional environmental benefits because of decreased greenhouse gas emissions and air and noise pollution (2). However, according to a study conducted during 2012–2019, less than 1% of people in the US achieve an average of 30 minutes per day of bicycling for any reason (3), and similarly, less than 1% of US workers bike to work (4).

The built environment plays a crucial role in promoting bicycle riding. Factors such as lack of infrastructure, increased car traffic, and resultant safety concerns inhibit bicycling (5,6). Conversely, infrastructure investments such as bike share programs and well-constructed bike lanes can improve safety and/or promote bicycling (7–10). Inequities in bicycling infrastructure investments exist between low-income and high-income communities in the US. Higher-income communities more often have resources dedicated to improving bicycle infrastructure as a means to promote physical activity, create livable communities, and foster economic growth, whereas plans, policies, and resultant infrastructure and projects that support bicycling are less common in communities with greater proportions of residents who have lower income and who are members of racial and ethnic minority groups (11,12).

Prioritizing equity in bicycle infrastructure, planning, and programming (IPP) through the use of a distributive justice lens and fair allocation of resources to lower-resourced neighborhoods is a promising way to promote access to bicycling opportunities (eg, bike lanes) among populations of low socioeconomic status (SES). This in turn can help reduce physical activity disparities, while also potentially achieving economic, social, and environmental co-benefits (13) in traditionally low-resourced communities. However, little is known about communities’ experiences with integrating equity in bicycle IPP at the local level. The objective of this project was to gain an understanding of the experiences, barriers, and facilitators that communities encounter with integrating equity considerations into bicycle IPP.

## Methods

This mixed methods study consisted of a national web-based survey followed by focus groups with a subsample of survey respondents. This project was determined not to be human subjects research by the University of Massachusetts Chan Medical School Institutional Review Board.

### The Bicycle Friendly Communities Program

Participants were people who represented communities across the US who had previously applied to the League of American Bicyclists’ (League) Bicycle Friendly Community (BFC) Program (14). The League is a national nonprofit organization that promotes bicycling as a safe, viable option for transportation and leisure activity for all. The BFC Program is an annual evaluation and recognition program that provides guidance to communities across the US on how to effectively increase bicycle ridership and safety through IPP. The BFC emphasizes the “5Es” (15) as a roadmap to accomplishing this: equity and access to bicycle-friendly environments for all, engineering that creates safe and convenient environments, education for the public to promote bicycling skills, encouragement through a welcoming culture, and evaluation and planning that includes bicycling as a transportation option. Applications are submitted on behalf of communities by designated individuals, such as transportation engineers, active transportation coordinators, and bicycling advocates.

### Survey

#### Design

We first conducted a cross-sectional survey to gather information on communities’ experiences with bicycle IPP activities designed to promote equity. The survey was administered in Survey Monkey in March, April, and May 2022.

#### Measures

The survey, designed by the study team, asked respondents about their community’s experiences implementing activities intended to foster equity in bicycling. The survey assessed 31 items in 3 activity categories: 1) community engagement, education, events, and programming (community engagement [10 items]); 2) data, evaluation, and goal setting (data [12 items]); and 3) infrastructure, facilities, and physical amenities (infrastructure [9 items]) (Table 1). Respondents could select an “other related activities” option in each category. A checklist format was used, and respondents endorsed the activities their community had ever implemented.

**Table 1:** Experience With Equity-Related Bicycle Infrastructure, Planning, and Programming Initiatives Among Respondents (N = 194) to a Survey Administered by the BFC Program Survey, March–May 2022

Experience (no. of survey items)	No. (%) of respondents^a^
**Community engagement (n = 10)**	
Bicycle classes, etc. Hosted certain affinity and identity groups	80 (41)
Free and subsidized access to bike classes and learning opportunities for low-income residents	69 (36)
Public outreach and engagement initiatives have intentionally focused on increasing equity	61 (31)
Translation services made available for bicycle-related outreach methods	47 (24)
Free learn-to-ride classes for adults with bikes available for participants	44 (23)
Bicycle-related survey or public meetings offered in languages other than English	43 (22)
Bicycle safety classes, etc. offered in languages other than English	42 (22)
Translation services made available for bicycle safety classes, etc.	21 (11)
Limited English Proficiency Plan and similar plans for bicycle engagement, etc.	8 (4)
Other similar effort	16 (8)
None of the above	36 (19)
Mean (SD) no. of items	2.2 (2.4)
**Data, evaluation, and goal setting (n = 12)**	
Socioeconomic or demographic data mapping incorporated in planning	69 (36)
Equity-focused analysis as part of a community planning effort and document	59 (30)
Intentional reparative or equity-focused transportation investments	47 (24)
Bicycle ridership, satisfaction, or safety data collection	45 (23)
Equity-related goals or performance measures have been established or adopted	33 (17)
Efforts to measure, identify, and eliminate racial disparities in traffic law enforcement	33 (17)
Community has joined the Government Alliance on Race & Equity or a similar entity	26 (13)
Community has adopted a Racial Equity Action Plan or similar	21 (11)
Officially recognized Transportation Equity Committee or similar advisory committee	19 (10)
Community has adopted an Inclusive Mobility Action Plan or similar accessibility	9 (5)
Anti-displacement program or strategies that relate to transportation investment	6 (3)
Other similar effect	22 (11)
None of the above	36 (19)
Mean (SD) no. of items	2.0 (2.3)
**Infrastructure, facilities, and physical amenities (n = 9)**	
Infrastructure or placemaking projects that intentionally reflect history of community	73 (38)
Free or subsidized access to bicycle safety equipment or accessories for low-income residents	71 (37)
Free or subsidized access to bicycles or bike share memberships for low-income residents	55 (28)
Adopted bicycle infrastructure design guidelines that specifically incorporate equity	33 (17)
Equity-related goals or performance measures have been established	32 (16)
Accessibility audit or assessment conducted for on- or off-road bike infrastructure	24 (12)
Free or subsidized access to adaptive cycles through bike share or other programs	16 (8)
Accessibility audit conducted for public bike parking	3 (2)
Other similar effort	25 (13)
None of the above	28 (14)
Mean (SD) no. of items	1.7 (1.7)
**Mean (SD) no. of equity-related activities (of 31)**	5.9 (5.7)

Abbreviation: BFC, Bicycle Friendly Community.

a Unless otherwise indicated.

#### Recruitment

The BFC director emailed an invitation to participate in the survey to all contact people listed on BFC applications from 2012 through 2021. After the initial email was sent, up to 3 emails were sent during 3 weeks to nonrespondents. A link to the survey was included in the email invitation and reminders.

#### Analysis

We used frequency distributions to indicate the percentage of respondents who had experience with each activity. Means and standard deviations were computed for all activities individually, for each of the 3 activity areas, and overall to evaluate experiences. We used Stata version 16 (StataCorp LLC).

### Focus groups

#### Design

We conducted 6 focus groups via Zoom in July and August 2022. Each focus group lasted approximately 90 minutes. Groups were led by an experienced facilitator (S.L.) and observed by a research team member (M.G., J.A.P.) who took notes and managed Zoom logistics.

#### Recruitment

At the end of the survey, respondents were asked if they were interested in being contacted to participate in a focus group discussion about their experiences integrating equity in bicycle IPP into their communities and if so, to provide their contact information. Of the 194 survey respondents, 70 agreed to be contacted. To enroll representatives of communities with different degrees of experience integrating equity into their bicycle IPP, we divided these respondents into 2 groups: a group with a high level of experience (at or above the median number of endorsed activities) and a group with a low level of experience (below the median number of endorsed activities). We created SignUp Genius calendars with several potential focus group times for the 2 groups and emailed messages to respondents in each group with instructions to indicate potential times they would be able to participate. Focus group times with the most signups were selected, and participants were notified by email of the time of the focus group they would participate in. The target number of people for each focus group was 6 to 8. This target number was chosen to allow for a group size that was small enough to foster an inclusive atmosphere yet large enough to generate a variety of responses and experiences. Once 6 groups (3 high experience, 3 low experience) were filled, we stopped enrolling. Participants who were allowed to accept an incentive for their participation (nongovernment employees) received a $50 gift card.

#### Focus group guide

We used a semistructured guide to facilitate discussions. The guide helped the facilitator describe the purpose of the focus groups and inform participants that the information gleaned would be used by the BFC program to inform development of its application and its work supporting BFC recipients. Definitions of bicycling and equity were provided to promote common understanding and clarify that participants should think about these concepts broadly and in a manner that is most relevant for the communities they serve. Bicycling was defined as including any kind of nonmotorized cycle (eg, adaptive cycles, recumbents, hand cycles, ebikes) for any purpose. Equity was defined as being inclusive of race, ethnicity, income, ability, geography, gender identity, sexual orientation, or any other characteristic. Questions were asked of participants about their experiences with integrating equity considerations into bicycle IPP, what has worked well with these efforts, lessons learned and what could be improved, and recommendations for additional supports to advance equity-focused work. Participants were also provided the opportunity to share any related thoughts that were not addressed in the guide.

#### Focus group analysis

Focus group discussions were recorded and transcribed in Zoom. Transcripts were de-identified and reviewed against recordings for accuracy. We used an established, validated rapid qualitative analysis methodology (16,17). Team members created a template of domains based in Excel (Microsoft Corp) on an initial review of the transcript that was used to summarize each focus group discussion. Two team members (M.G. and J.A.P.) summarized the first focus group independently and discussed findings to ensure consistency and completeness before summarizing the rest of the groups. Then, a single team member (M.G. or J.A.P.) completed coding for each remaining group. Coders met biweekly to discuss progress, make refinements to the template and the process as needed, and review each other’s summary templates. Coders discussed perspectives on emerging themes and determined if or when saturation was met. The team created a matrix from all 6 focus groups, and from this matrix, reviewed and synthesized domains into themes. Themes that emerged were consistent with the 3 survey content areas and were organized as such. We compared the results of high- and low-experience groups. All focus group participants were provided with an initial summary report of findings and given the opportunity to provide input on whether the results mirrored their experiences (18).

## Results

### Survey

The survey was completed by 194 respondents (of 685 invited; 28.3% response rate) located in 44 states and the District of Columbia. The median size of the communities in which survey respondents worked was 65,098 people (IQR, 25,097–169,467). Respondents reported experience with a mean (SD) of 5.9 (5.7) activities of 31 equity-related activities overall; 2.2 (2.4) of 10 activities related to community engagement, education, events, and programming; 2.0 (2.3) of 12 activities related to data, evaluation, and goal setting; and 1.7 (1.7) of 9 activities related to infrastructure, facilities, and physical amenities (Table 1).

### Focus groups

The 6 focus groups included 30 participants representing 17 states. Most (23 of 30) were city or county employees, with the remainder representing advocacy organizations. The median size of the communities in which focus group participants worked was 73,500 people (IQR, 32,187–183,772). Focus group participants reported more experience than the survey sample with equity-focused activities (mean, 9.0; SD, 5.5 of 31 activities). We observed no substantive differences between the high-experience and low-experience groups; thus, we combined the results on themes and associated barriers and facilitators organized by survey content area (Table 2).

**Table 2:** Results of 6 Focus Groups on Themes and Associated Barriers and Facilitators of the BFC Program Survey, by Survey Content Area, and Illustrative Quotes of Participants (N = 30), July–August 2022

Survey content area	Quotes About Barriers	Quotes About Facilitators
**Community engagement**		
Outreach and engagement of individuals from low SES communities	• How do we develop these projects and partnerships so you know they’re seen as responding to community needs as opposed to what kind of downtown planners and engineers might want to happen? • We rely heavily on advocacy organizations and if there isn’t an organization that addresses — that represents — an underserved community, then we don’t know how to reach out to them. • If they don’t trust us in the first place, then they’re not going to really even bother responding, so they’re not represented in those things.	• Having the organization look like the people we serve is probably the single best way to get successful outcomes. • We created paid positions that are called neighborhood leaders . . . for the different neighborhoods, and in a lot of cases, these are already people who are established in those neighborhoods who people trust, and they can be that conduit. • The new inclusion office and that role really was helpful to facilitate conversations with the community and be kind of a connection between the community and the government.
Cultural perceptions of bicycling	• We’re struggling to help people understand that biking programs and infrastructure have widespread benefits to people of all walks of life. • People who cannot afford a car are [perceived as] the ones to ride bikes. • There’s a belief in our city that bike lanes are just for people in spandex.	• I think it’s really good that the league continues to represent, you know, Black and Brown people in a lot of their literature and their publications and the website. It is important to see that there are people who look like me who ride bikes, you know, and who are interested in bicycle planning. • Representing the diverse spectrum of what cycling means — I think there is power in having graphics that show, like, everyone’s not the same. . . . [I]maging that makes people feel like they’re included in the movement as well.
Funding and support for community rides and programs	• It’s applauded by the city, but I wouldn’t say supported. • People agree to [ideas] until it is time for them to commit dollars, which is when they pull back. • We’re struggling to help people understand that biking programs and infrastructure have widespread benefits to people of all walks of life: disabled people, seniors, people who drive, people who need to park.	• Having those strong partners in our advocacy group, bike ped, and also our bike share and more nonprofits — those really help run those types of programs. Those [are] encouragement and education programs that we really can’t do at the city. • The [inhouse government outreach and marketing groups] are already connected to the community and they’re already great marketers and information spreaders, and so we were able to funnel money into helping with basically the salaries to get hours of work toward our marketing and outreach.
**Data**		
Availability of locally relevant data	• National definitions of equity did not meet our local conditions. Like the census tract for a town our size is not the appropriate kind of planning level to evaluate. • I think one of our unmet needs . . . is that we don’t share across departments a definition of what it means when we’re doing equity work or what parts of the city or what populations, we would be working with.	• We have really good local data through our health department. • We have developed a prioritization model to prioritize projects, and equity is one of the factors in there, and we have a couple of different metrics related to income, race, [and] vehicle availability that are in there, so projects are scored. • For us, having things — policies — having a way that we prioritize things that has been approved by our Council. It’s, you know, that’s how we do things. It has been very good at trying to get away from the challenge that we continue to have, which is that it’s the loudest people who know how to get things done.
**Infrastructure**		
Political will, governmental collaboration, and understanding of bicycling needs	• There’s a lack of internal coordination [across departments] and kind of agreement on what the goals are for the town and then that we should all be working together on this. • Decision makers even though they might be bike-friendly don’t understand what it takes to get there. • People are buying into the concept of equity only at face value, without really diving into what it actually means, but then as soon as we dive deeper, it means possible resources and or inconveniences, there is resistance.	• We just had a town manager who had been here for over 10 years, and he was really a very good advocate, champion for biking within the town. And we were able to get some things through our public works that might not have otherwise happened if he hadn’t been there. • Helping our elected officials and decision makers better understand the connections between bikeability and economy, jobs, sustainability, equity, health, and safety. • Involvement is needed in large numbers through letter writing and public comment to influence political will.
Community design and infrastructure	•There’s just so many factors that go into infrastructure and in older parts of town or areas that tend to lack infrastructure, they get overlooked. . . . [T]hings that are aging are obviously much more expensive, and so they tend to get pushed aside. • We struggle with getting anything done that doesn’t look like it’s “always looked” and a belief that bike infrastructure is “big city stuff.” • You know, our legislature is very conservative [and] our city is fairly progressive, and so anytime we try and do something to make life easier, our legislature, sometimes just takes action, just to spite us.	• In our applications, we have to demonstrate certain equity, a project meets certain equity requirements. • One of the successes that we’ve had and that I found really helpful, is we are a Complete Streets community, so in order for us to receive, you know, state funding that trickles down, we have to have a Complete Streets policy. • The new BFC [Bicycle Friendly Community] application and the equity measures are going to drive real change here. Because all we have to say is we need this to get gold because we’re silver now . . . and people said, “Oh well, we got to do that.”

### Community engagement

Three themes related to community engagement emerged: 1) outreach and engagement of members of low SES communities, 2) public education and cultural perceptions of bicycling, and 3) funding and support for community rides and programs for communities of low SES.

Outreach to and engagement of members of low SES communities was something most participants had conducted. However, few reported successes in these efforts. Participants voiced a general lack of confidence in their ability to reach communities of low SES and reported that lack of trust by community members was common. Most had relied on members of their departments or advocacy organizations to conduct these efforts, and these people were rarely representative of the communities of focus. Although a few participants indicated identifying or having funding to address this gap, some participants reported success with strategies that allowed engagement with communities, such as hiring outreach specialists or community champions and collaborating with government equity offices, which were described as newly emerging in some communities. These strategies allowed for bidirectional communication and community input into their equity-focused work.

Cultural perceptions of bicycling were commonly discussed. Community stigma, that only people who cannot afford a car ride bicycles, particularly to work, was commonly reported by participants as a barrier to addressing bicycling equity. However, the perception of bicycling as an elite activity only for “people in spandex” was also prevalent, resulting in challenges to effective messaging. Several participants identified educational campaigns featuring relatable role models from diverse racial and ethnic backgrounds, provided in multiple languages, as a promising educational strategy. However, few communities had conducted such campaigns.

Funding and support for community rides and programs for communities of low SES was described frequently by participants. They noted that funders, government, and the community did not provide sufficient support for these types of activities because they did not perceive a demand or interest in them. Lack of capacity of bicycling trainers to work with equipment for people with disabilities, such as recumbent bikes, was noted. Despite these challenges, participants described various programmatic efforts aimed at integrating equity, such as distribution of helmets and maps, providing free tune-ups at community events, and offering educational programs. Successful strategies for reaching communities of low SES with community rides and programs included strong collaborations with advocacy groups, local bike shops, and other groups to offer trainings, equipment, and repairs, and leveraging the marketing infrastructure in local government for promotion to the community of focus.

### Data

Availability of locally relevant data to inform infrastructure planning and programing was universally described as important. The need for local, neighborhood-level data to educate decision makers and to inform prioritization of initiatives and funding was well-recognized. Various measurement approaches were used. However, these data were limited because they were not usually geographically relevant. Census-based data sources did not often overlap with neighborhood geography or the sociodemographic composition of the community. Some participants described a lack of consensus on what defined equity and a lack of coordination across government departments, all of which relied on neighborhood-level data, albeit with different priorities. Facilitators to collecting relevant primary data included ordinances calling for equity-based data-driven prioritization, cross-department collaboration that involved leveraging staffing and funding resources, and the ability to use the BFC application tool to educate local officials on the need to prioritize equity.

### Bicycle infrastructure

Two infrastructure-related themes emerged: 1) political will and governmental collaboration, and 2) challenges with community design and infrastructure in low-income areas.

Limited political will, governmental collaboration, and understanding of bicycling needs was commonly discussed. Participants generally described a lack of collaboration across local governmental departments to prioritize integrating equity considerations into planning activities in general and for bicycle infrastructure specifically. They also described a lack of understanding and interest among high-level decision makers on the need to prioritize low-income neighborhoods for community design improvements. Participants reported needing to help decision makers make connections between equity-focused infrastructure planning and areas they typically prioritize, such as economic development, and needing to increase community advocacy. Even though having a government champion (eg, mayor) was a recognized facilitator to supporting cycling in general and equity efforts in particular, this facilitator was uncommon.

Community design and infrastructure was a key point of discussion. Participants described aging and poorly designed infrastructures as common in low-income communities. Because these communities were often more expensive and challenging to redesign, they were often not prioritized. Challenges with prioritizing equity-based infrastructure improvements were more common among participants who identified themselves as being from politically conservative states. Increased adoption and recognized value of policies such as the US Department of Transportation’s Complete Streets (19) policies were described as helpful and often necessary for prioritizing planning and transportation initiatives in low-income neighborhoods.

## Discussion

In this mixed methods study of participants in the League’s BFC program, participants indicated a modest level of experience with activities that supported the integration of equity considerations into bicycle IPP. Our findings suggest that the barriers and facilitators to incorporating equity into bicycling IPP are fairly universal across US communities. Highly experienced communities, compared with those with a low level of experience, typically had been working toward equity for a longer time or had more resources to implement potential solutions. However, focus group participants universally supported its importance, and a shift in momentum toward prioritizing equity in their work was clear. Despite noted barriers related to community engagement, data, and infrastructure, participants highlighted promising strategies.

About one-third of survey respondents reported activities focused on outreach to and engagement of communities. Focus group participants widely recognized the importance of these efforts to build support for focusing bicycling IPP to benefit communities. However, efforts had not been appropriately resourced and, thus, relied on existing staff or bicycling advocacy organizations. In addition to limited involvement in traditional public engagement opportunities for transportation planning projects, members of low SES and minority populations are underrepresented in the planning profession and bicycling advocacy organizations (20). A need remains for true community engagement to build public support for equity-focused bicycling IPP. Focus group findings offered suggestions for maximizing outreach and engagement, such as hiring dedicated staff who represent communities of focus and partnerships with local community-driven groups that concentrate on other equity-focused topics, to work toward bidirectional approaches.

The discussions revealed somewhat conflicting perspectives on cultural barriers to bicycling. On one hand, participants reported that their communities experienced stigma that bicycling is an indicator of low SES (ie, people who can’t afford a car are bicycling out of necessity). On the other hand, they expressed the idea that bicycling is only for the elite “spandex” crowd (21). Recommended approaches to addressing these perspectives centered on promoting social norms through public health campaigns that highlight diverse community members bicycling. Although these are likely worthwhile efforts, we note that focus group discussions focused more on individual-level factors than on broader systemic issues, particularly systemic issues experienced by racial and ethnic minority populations, that drive community concerns about bicycling (as well as walking) for active transportation (18,21). Factors rooted in racism, such as high rates of ticketing among racial and ethnic minority community members while bicycling, fear of police brutality in public spaces, and higher bicycle fatality rates in low-income communities, must be recognized in all efforts to promote bicycling among racial and ethnic minority populations (22).

Most communities had some level of experience related to the use of data, metrics, and strategic goal-setting in bicycling IPP. The most common activity reported in the survey was mapping socioeconomic or demographic data in planning activities. Likewise, focus group discussions focused on the need to use data for advocating to decision makers, prioritizing projects, and evaluating impacts. Our study participants relied largely on existing census-based measures to determine which communities should be prioritized for equity-focused initiatives because of the convenience and low cost of these measures. However, these secondary data sources often did not capture data at a unit of geography that overlapped with neighborhoods or communities in their jurisdiction, making it challenging to effectively use such data for prioritization. Consensus across government departments or with community input on how to define local priority neighborhoods and communities based on geography or other metrics, along with aligned efforts to collect primary data on community-specific metrics that could inform bicycling infrastructure improvements, were uncommon but also indicated as necessary for positing and supporting strategic investments. Successful examples include cities, such as Denver and Atlanta, that have adopted and implemented a common measurement approach that guides prioritization (23,24).

The existing physical infrastructure in low-income communities was a noted barrier to bicycling infrastructure improvements. These neighborhoods were noted to be older and more poorly designed than higher-income neighborhoods, conditions that are rooted in historical injustices and ongoing lack of equitable investments in neighborhood revitalization (18). Planners in particular described expenditure, coupled with lack of political will, as key barriers. Despite these challenges, examples of successful bicycling infrastructure improvements, with and without other investments in the built environment and economic development of low-income neighborhoods, provide guidance on successful approaches (25,26). The Watts Rising initiative in Los Angeles is such an example (27). This broad-based community and data-driven initiative entails multisector collaboration (eg, housing authority, local elected officials, community organizations) to improve quality of life through infrastructure investments that promote health, economic development, and environmental improvements, including bicycling infrastructure. This example illustrates the need for investing in community engagement to drive advocacy within communities and build long-standing, trusting relationships among communities, organizations, and decision makers to enable infrastructure changes and redesign communities.

Lack of political will to prioritize bicycling overall and in low-income neighborhoods in particular was commonly reported by focus group participants. This barrier was particularly striking in local communities identified as more politically liberal in states that were more traditionally conservative. Having a champion at high levels of local government, noted by participants, has been effective in promoting active transportation infrastructure (26). A champion may arise serendipitously or require careful cultivation. Making the case for resources to invest in infrastructure based on co-benefits is more likely to appeal to decision makers. For example, the economic development associated with community redesign (28) was noted by focus group participants as a potential facilitator and is consistent with contemporary thinking about effective public health messaging approaches for policy, systems, and environmental interventions. Community members, advocates, planning officials, and allied government officials likely need training and support to effectively develop and articulate these messages to decision makers. The BFC application tool, which was revamped in 2022 to incorporate equity-focused activities as a key criterion, was highlighted as an important tool to assist with this messaging.

Despite being provided an inclusive definition of equity, focus group participants largely focused their discussions on equity pertaining to neighborhood income level, race, and ethnicity. Discussions on equity pertaining to physical mobility, whether related to disability or age, were largely limited to providing appropriate equipment and offering training. Participants reported struggling with the capacity to offer such services and expressed the need for both funding and staffing to offer them. Little was mentioned about infrastructure or planning activities that addressed the requirements of community members with different mobility needs. This oversight may have been due to factors such as focusing on neighborhood-level approaches to decision-making and resource investments or lack of data to guide prioritization. Regardless, inclusive community design, whether for bicycling or other forms of mobility, is rarely prioritized (29) and deserves more consideration from decision makers and advocates.

Information gleaned from this study has been used by the League in 2 major ways. First, findings informed a critical update to the BFC application in 2023, which now features a new section, Equity & Accessibility, as well as new equity-related question-and-answer options that have been integrated into the other sections of the application. This update accounts for approximately 22% of the points available for a community’s overall BFC score. Second, findings have been used to establish a baseline understanding of the frequency with which various equity-related practices are used, to better weigh and evaluate applicants’ responses to the new equity questions on the updated application. Applicant communities now also receive an updated BFC report card, which highlights their Equity & Accessibility section and subsection scores so that they can measure and track their progress on equity-related efforts.

### Strengths and limitations

This study has strengths and limitations. The mixed methods design allowed for a deeper understanding of our study topic than the use of a single methodology would provide. Partnership with the BFC program brought credibility to the research and guided the collection of action-oriented data that can be used to guide practice. The generalizability of findings, however, may be limited by the inclusion of participants representing communities, in both the survey and focus groups, that have more interest or experience with bicycle IPP than US communities in general. The study also did not capture participants’ sociodemographic information.

### Conclusion

To our knowledge, this study is among the first that seeks to understand communities’ experiences with activities that support integrating equity considerations into bicycle IPP and factors that promote and inhibit these considerations among government decision makers and advocates in a national sample. Although they recognized the challenges of equity-related work, participants emphasized moving toward approaches that prioritize equity. Findings yielded potentially actionable strategies that can support equity in bicycle IPP and reduce disparities in physical activity and associated health conditions.
